# Probing Corticospinal Control During Different Locomotor Tasks Using Detailed Time-Frequency Analysis of Electromyograms

**DOI:** 10.3389/fneur.2019.00017

**Published:** 2019-01-29

**Authors:** Linard Filli, Christian Meyer, Tim Killeen, Lilla Lörincz, Beat Göpfert, Michael Linnebank, Vinzenz von Tscharner, Armin Curt, Marc Bolliger, Björn Zörner

**Affiliations:** ^1^Department of Neurology, University Hospital and University of Zurich, Zurich, Switzerland; ^2^Spinal Cord Injury Center, Balgrist University Hospital, Zurich, Switzerland; ^3^Department of Biomedical Engineering, Center for Biomechanics and Biocalorimetry, University of Basel, Basel, Switzerland; ^4^Department of Neurology, Helios-Klinik Hagen-Ambrock, Hagen, Germany; ^5^Faculty of Kinesiology, University of Calgary, Calgary, AB, Canada

**Keywords:** neuromuscular control, locomotion, electromyography, humans, corticospinal, walking

## Abstract

Locomotion relies on the fine-tuned coordination of different muscles which are controlled by particular neural circuits. Depending on the attendant conditions, walking patterns must be modified to optimally meet the demands of the task. Assessing neuromuscular control during dynamic conditions is methodologically highly challenging and prone to artifacts. Here we aim at assessing corticospinal involvement during different locomotor tasks using non-invasive surface electromyography. Activity in tibialis anterior (TA) and gastrocnemius medialis (GM) muscles was monitored by electromyograms (EMGs) in 27 healthy volunteers (11 female) during regular walking, walking while engaged in simultaneous cognitive dual tasks, walking with partial visual restriction, and skilled, targeted locomotion. Whereas EMG intensity of the TA and GM was considerably altered while walking with partial visual restriction and during targeted locomotion, dual-task walking induced only minor changes in total EMG intensity compared to regular walking. Targeted walking resulted in enhanced EMG intensity of GM in the frequency range associated with Piper rhythm synchronies. Likewise, targeted walking induced enhanced EMG intensity of TA at the Piper rhythm frequency around heelstrike, but not during the swing phase. Our findings indicate task- and phase-dependent modulations of neuromuscular control in distal leg muscles during various locomotor conditions in healthy subjects. Enhanced EMG intensity in the Piper rhythm frequency during targeted walking points toward enforced corticospinal drive during challenging locomotor tasks. These findings indicate that comprehensive time-frequency EMG analysis is able to gauge cortical involvement during different movement programs in a non-invasive manner and might be used as complementary diagnostic tool to assess baseline integrity of the corticospinal tract and to monitor changes in corticospinal drive as induced by neurorehabilitation interventions or during disease progression.

## Introduction

Walking is a complex motor task requiring a high degree of coordination and balance. Different movement patterns are initiated and controlled by specific neural networks that orchestrate the coordinated activity of numerous muscles. Simple movements are thought to be primarily controlled by evolutionary ancient, subcortical central nervous system (CNS) areas (e.g., brainstem nuclei, basal ganglia networks, cerebellum), whereas skilled, fine-tuned movements, as required for adaptive and predictive motor actions, rely more on corticospinal commands (1–5). Our knowledge of neuromuscular control during walking is based predominantly on vertebrate animal models, with confirmatory evidence in human locomotion notably lacking (4, 6).

Probing neuromuscular control in humans is methodologically challenging: functional magnetic resonance imaging and magnetoencephalography are susceptible to movement artifacts, thus limiting their use under dynamic conditions (7, 8). Positron emission tomography requires radioactive tracers and is thus problematic for longitudinal assessment. Electroencephalography is difficult to interpret and provides only limited spatial resolution of brain activity (9). Functional near-infrared spectroscopy is increasingly used in the field of neuroscience, however, its application protocols are not yet standardized, complicating comparison of data between research centers (7). Transcranial magnetic stimulation is difficult to perform under dynamic conditions, requiring distracting neural stimuli.

Surface electromyography is an important non-invasive technology for monitoring neuromuscular control during dynamic tasks (10), containing information that may be used to partially decode the neural drive underlying neuromuscular control (11). Classically, electromyograms (EMGs) are analyzed in the dimensions of time and amplitude. The use of highly resolved intensity patterns allows resolution of an EMG's intensity (square root of power) in time and frequency (12). Robust evidence from corticomuscular coherence experiments in humans suggest that corticospinal drive to muscles can be gauged by the extent of synchronous oscillations in the frequency range of the Piper rhythm (13–20). Motor unit action potentials that occur almost simultaneously in this rhythm can also be measured between different muscles (intermuscular coherence) and have been associated with shared corticospinal commands controlling voluntary movements (17, 21). Enhanced synchrony of EMG signals in the Piper rhythm frequency was observed during challenging locomotor tasks that strongly rely on cortical control (22, 23). In addition, EMG intensity in this frequency range was significantly reduced in patients with stroke (24, 25), spinal cord injury (26) and spinocerebellar ataxia (27), suggesting the potential role of spectral EMG analysis as biomarker for corticospinal integrity (17). These results imply that time-frequency resolved electromyography is able to gauge corticospinal drive during dynamic conditions in the absence of direct measures of cortical activity. The origin of EMG intensity at frequency ranges other than the Piper rhythm is poorly understood (16), but might represent the activation of different slow- and fast-conducting muscle fibers during a given motor task (20, 28).

The purpose of this study is to examine neuromuscular control during various walking tasks that were chosen to place varying demands on the neuromuscular system. Changes in neuromuscular control were assessed by analyzing EMGs of distal leg muscles during the different walking tasks. We hypothesize that neuromuscular control of skilled walking, using targeted stepping, differs most from regular walking with respect to cortical input, and thus induces specific changes in time-frequency resolved EMG intensity reflective of enhanced corticospinal control. Non-invasive, widely-used EMG analysis might be an attractive method supplementing common clinical measures of corticospinal integrity (e.g., transcranial magnetic stimulation, MRI-based imaging etc.): time-frequency resolved electromyography might be used to gauge initial corticospinal impairment (25, 27), as well as to monitor changes in pyramidal tract function in the background of disease progression or in response to different therapeutic approaches (21). A better understanding of the neural mechanisms underlying different motor tasks of everyday living (e.g., locomotion) might guide the development of optimized, tailored rehabilitation programs for individual patients.

## Materials and Methods

### Participants

All study participants were assessed at the University Hospital Zurich and were recruited via flyers from the local area. The participants underwent detailed medical screening to exclude any orthopedic or neurological abnormalities confounding locomotor analysis (including color-blindness). This study was approved by the Zurich cantonal ethics committee (KEK-2014-0004) and was conducted in accordance with the Declaration of Helsinki. All subjects gave written, informed consent.

### Study Design and Experimental Procedures

During the first visit, all participants completed an acclimatization protocol of 30–40 min duration to familiarize themselves with the treadmill (120 Hz, FDM-T, Zebris Medical GmbH, Germany), and the different locomotor conditions. Within 7 days of the first visit, all participants returned for a second visit, during which the walking tests were performed and EMG and kinematic data were recorded. Besides regular walking, participants walked while performing a distracting, cognitive dual task, while wearing protective goggles obscuring the lower half of the visual field (visual restriction), and while targeting crosses projected onto the moving treadmill belt (targeted walking). Participants walked while fixing their gaze onto a screen (22″ LCD monitor) positioned at eye height in front of and facing the treadmill. The dual task consisted of two versions of a modified Stroop task [word-color discrimination test (29)] displayed while walking. In the congruent (easy) dual task, the meaning and color of the displayed word were identical. In the incongruent (difficult) condition, the word and color were in conflict, enhancing cognitive loading. For both dual task conditions, participants were instructed to name the color of the word as accurately and quickly as possible. Words on the screen were shown in pseudorandom intervals (600–1,400 ms between words; ≥200 ms difference between consecutive intervals) to obviate potential rhythmic cuing of the walking pattern. In a third condition, participants walked with the lower half of their visual field obscured by modified protective goggles (30). Removal of direct visual feedback from body movements likely enhances somatosensory and vestibular contributions to locomotor control (31, 32). The goggles were adjusted such that the visual field inferior to the interpupillary line was obscured. During visually guided, targeted walking, participants looked at the floor and tried to target and step on moving crosses (100 × 100 mm) projected onto the treadmill. This information was reported above. Mediolateral and anteroposterior intercross distances were irregular and were calculated based on maximal stride length and step width of each individual (varying randomly between 40 and 80% of each participants' maximal stride length and step width). Participants performed all locomotor trials while walking on the treadmill at half-maximal walking speed (v_max50%_) as determined in a timed 25-foot walk (T25FW) test. Additionally, participants performed the regular walking task at a fixed, slow speed (1 km/h).

Instrumented gait analysis was performed while participants walked with a stable pattern for >30 s. For kinematic analysis, participants were equipped with 39 reflective markers (14 mm diameter; modified Cleveland model) detected by 14 infrared cameras (Vicon, Oxford, UK; 200 Hz sampling rate). Bipolar Ag-AgCl surface electrodes (type Blue Sensor N, Ambu A/S, Denmark) were placed bilaterally on the tibialis anterior (TA) and gastrocnemius medialis (GM) muscles (20 mm inter-electrode distance) in accordance with the European recommendations for surface electromyography [SENIAM (33)]. The skin was shaved, lightly abraded (Nuprep gel, Waver and Company, USA) and cleaned with alcohol prior to electrode placement. Cables and wireless transducers were fixed to the skin with tapes and leg stockings (Leggyfix; TYTEX, Denmark) to minimize movement artifacts. EMGs were recorded at 1,000 samples/s and amplified by a factor of 1,000 using a 12-channel wireless Myon 320 system (Myon AG, Schwarzenberg, Switzerland) with a bandwidth range of 5–500 Hz.

### Data Processing

Kinematic data was processed in Nexus 2.2.3 (Vicon, Oxford, UK) and gait events were defined by zero-crossings in heel marker velocity as described previously (34, 35). Continuous EMGs of left and right TA and GM muscles were cut (heel strike to consecutive ipsilateral heelstrike). The quality of EMGs was assured by visual inspection: one principal evaluator thoroughly investigated raw signals and rectified EMGs on the basis of each step cycle, all participants and each walking condition. As walking patterns and EMG signals of healthy individuals are highly consistent and repetitive, non-physiological signal artifacts were easily identified in the majority of cases. Only EMG signals revealing clear-cut artifacts were removed from analysis to obviate confounding effects on the results. Ten or more gait cycles per walking condition per subject were required for a muscle to be analyzed. EMGs of the left and right leg were analyzed. Data for 2 left and 2 right TA muscles and 9 left and 6 right GM muscles showed aberrant EMGs and were excluded from further analysis. In subjects with unilaterally aberrant EMGs, only the contralateral EMG signals were included in the analysis.

Raw EMGs for single gait cycles were decomposed into time and frequency EMG intensity components using proEMG software (prophysics AG, Switzerland) employing a non-linear wavelet transformation algorithm. The wavelet transformation yielded the intensity (square root of power) of the EMG resolved in nine frequency bands by center frequencies 7, 19, 38, 62, 92, 128, 170, 218, and 271 Hz. The EMG intensity for each stride was time-normalized to 100% and the average of the normalized EMG intensities of all strides was computed for each walking trial (12, 36, 37). For each subject and condition, this resulted in a data matrix of 100 time points × 9 wavelets, where the abscissa represents the normalized time point in gait cycle, the ordinate the frequency of signal and the coloring the intensities of the signal. Summing the intensities at each time point gives the total intensity, whereas summing all intensities at particular frequency gives the wavelet intensity spectrum.

Raw EMGs of GM obtained during regular and targeted walking were additionally submitted to a fast Fourier transformation (256 samples, starting from muscle activity onset). The power spectra (3.9 Hz resolution) of 3 step cycles were averaged per subject and the standard error of the mean estimated.

### Statistical Analysis

Statistical analysis was performed using SPSS (V25, SPSS Inc., CA, USA). Assumptions of normality and homogeneity of variance were ascertained by the Kolmogorov-Smirnov test and the Levene's test. Area under the curve, representing total intensity and mean frequencies during the different walking conditions were compared using a 1-way repeated measures ANOVA followed by Dunnett's *post-hoc* correction for multiple comparisons. Total EMG intensity and EMG intensity within different frequency bands were analyzed by repeated measures 2-way ANOVA with two independent, within-subject variables (see figure legends for information on variables used in particular tests). Dunnett's *post-hoc* correction was applied to analyze the effect of locomotor conditions on EMG intensity at particular time points (within the gait cycle) and at specific frequency ranges. Comparison of area under the curve representing total intensity and mean frequency between half-maximal walking speed and 1 km/h gait speed was done using two-tailed, paired *t*-tests. The level of significance was set at 0.05 for all tests.

## Results

### Study Population

Thirty-three healthy participants were screened for study eligibility. Three were excluded due to a history of lower limb or spine surgery and one subject terminated the study prematurely. Two participants were excluded from the analysis due to failure in EMG recordings. EMGs of TA and GM muscles were analyzed in 27 participants (11 females; mean ± SD: 48.9 ± 9.6 years; 76.6 ± 19.4 kg; 172.7 ± 11.4 cm). Mean half-maximal walking speed was 4.3 ± 0.6 km/h.

### Time-Amplitude Characteristics of EMG Intensity Differs During Various Locomotor Tasks

**Figure 1:**
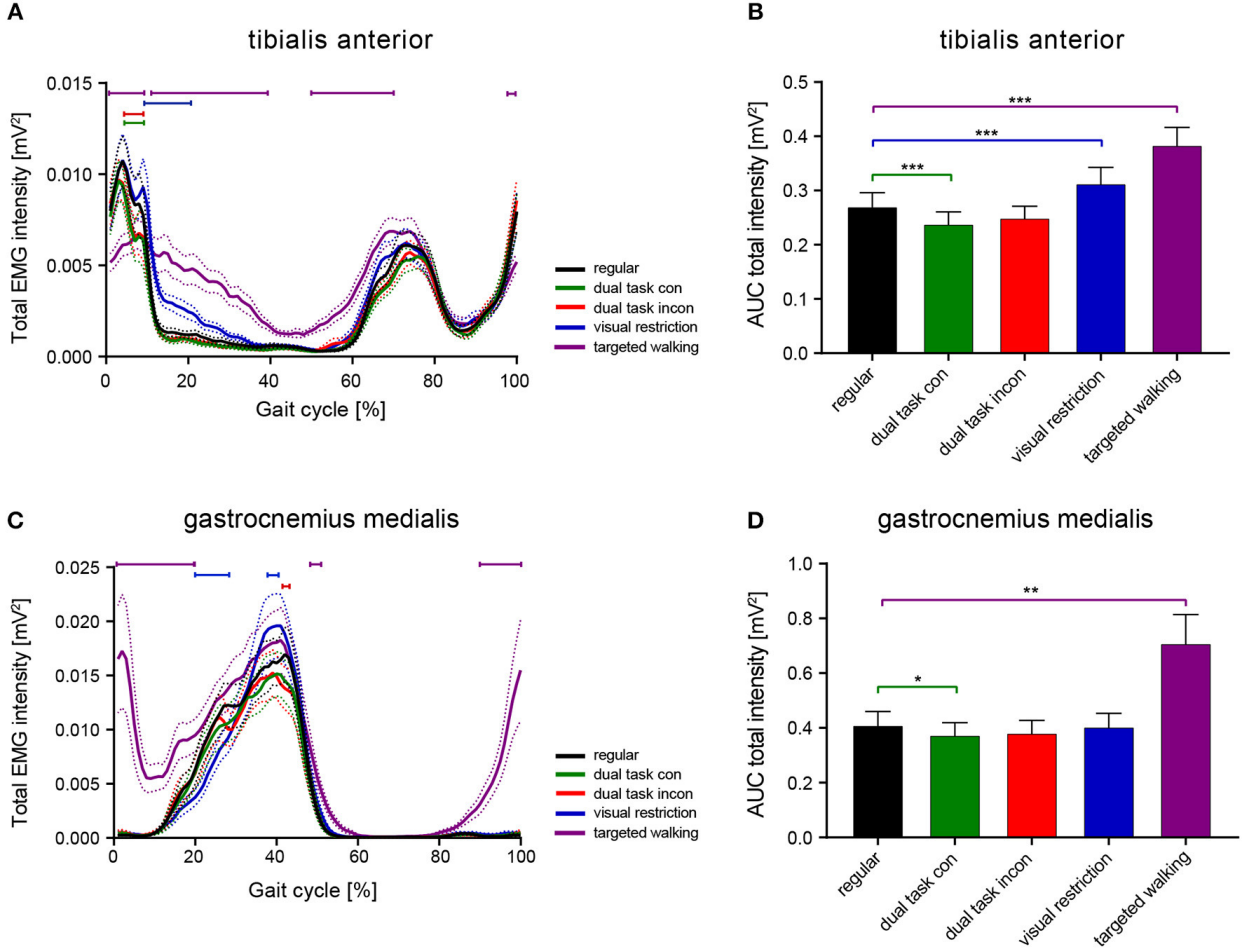
Total EMG intensity resolved in time and amplitude during various locomotor tasks. Averaged total EMG intensity and area under the curve (AUC) representing EMG intensity of the TA **(A,B)** and GM muscle **(C,D)** during various walking conditions. Statistical analysis of total EMG intensity over time was performed using repeated measures 2-way ANOVA with the independent factors time (within the gait cycle) and locomotor conditions. AUC data were analyzed with 1-way ANOVA repeated measures. Detailed effects of locomotor tasks on total EMG intensity **(A,C)** and AUC **(B,D)** were examined by Dunnett's *post-hoc* correction for multiple comparisons. *P < 0.05; **P < 0.01; ***P < 0.001. AUC, area under the curve; con, congruent; incon, incongruent; EMG, electromyogram; GM, gastrocnemius medialis; TA, tibialis anterior.

We analyzed the effects of the factors locomotor conditions and time (within the gait cycle) on EMG intensity of TA and GM (repeated measures 2-way ANOVA with within-subject factors time and locomotor conditions) and found significant main effects for locomotor conditions [TA: *F*_(4, 196)_ = 21.56; *p* < 0.0001 and GM: *F*_(4, 152)_ = 14.1; *p* < 0.0001] and time [TA: *F*_(99, 4, 851)_ = 37.98; *p* < 0.0001 and GM: *F*_(99, 3, 762)_ = 38.19; *p* < 0.0001] with a significant interaction effect [TA: *F*_(396, 19, 404)_ = 8.79; *p* < 0.0001 and GM: *F*_(396, 15, 048)_ = 6.59; *p* < 0.0001]. *Post-hoc* analysis of walking condition effects on EMG intensity patterns revealed that walking while performing the cognitive dual tasks (congruent and incongruent Stroop tasks) led to a reduction in TA EMG intensity during early stance phase compared to regular walking (Figure 1A; 4–9% of gait cycle; *p* < 0.05; repeated measures 2-way ANOVA followed by Dunnett's *post-hoc* correction for multiple comparisons). The timing of the two initial muscle intensity peaks was not affected by cognitive interference. Time-amplitude characteristics of TA EMG intensity were not different between the congruent (easy) and incongruent (difficult) Stroop test. Lower visual field restriction resulted in more prolonged TA activity after initial foot contact (9–21% of gait cycle; *p* < 0.05). The highest deviations in TA EMG intensity from regular walking were found during skilled, targeted walking: whereas EMG intensity of the TA was reduced before and after initial foot contact (98–100 and 1–9% of gait cycle; *p* < 0.05), it was enhanced during midstance phase (11–39% of gait cycle; *p* < 0.05) and during stance to swing transition (Figure 1A; 50–70% of gait cycle; *p* < 0.05). EMG intensity in the GM muscle revealed only brief alterations brought about by cognitive distraction during walking (Figure 1C; incongruent Stroop task: 42–43%; repeated measures 2-way ANOVA followed by Dunnett's *post-hoc* correction for multiple comparisons; *p* < 0.05). In contrast, visual restriction induced time-delayed GM activation (20–28%; *p* < 0.05) and enhanced peak EMG intensity (37–43%; *p* < 0.05) during stance. Similar to TA, targeted walking induced the most prominent deviations from normal GM physiology: EMG intensity was strongly enhanced before and after initial foot contact (90–100 and 1–20% of gait cycle; *p* < 0.05) and peak intensity during stance was increased (48–51%; *p* < 0.05).

Integrated EMG intensity (area under the curve, AUC) was changed for both TA and GM in response to different walking conditions [TA: *F*_(1.717, 84.15)_ = 22.25; *p* < 0.0001; GM: *F*_(1.086, 41.26)_ = 14.16; *p* = 0.0004; repeated measures 1-way ANOVA]. *Post-hoc* analysis revealed reduced AUC of the TA during the congruent dual task condition (*p* = 0.0008; repeated measures 1-way ANOVA followed by Dunnett's *post-hoc* test) and enhanced AUC during visual field restriction (*p* = 0.0003) and targeted walking (*p* = 0.0002) compared to regular locomotion (Figure 1B). Integrated EMG intensity of the GM revealed reduced muscle activity during the congruent cognitive task (*p* = 0.0324; repeated measures 1-way ANOVA followed by Dunnett's *post-hoc* test), and enhanced values for targeted walking (*p* = 0.0024; Figure 1D) compared to regular walking.

### Frequency Characteristics of EMG Intensity Are Modulated Most by Targeted Locomotion

**Figure 2:**
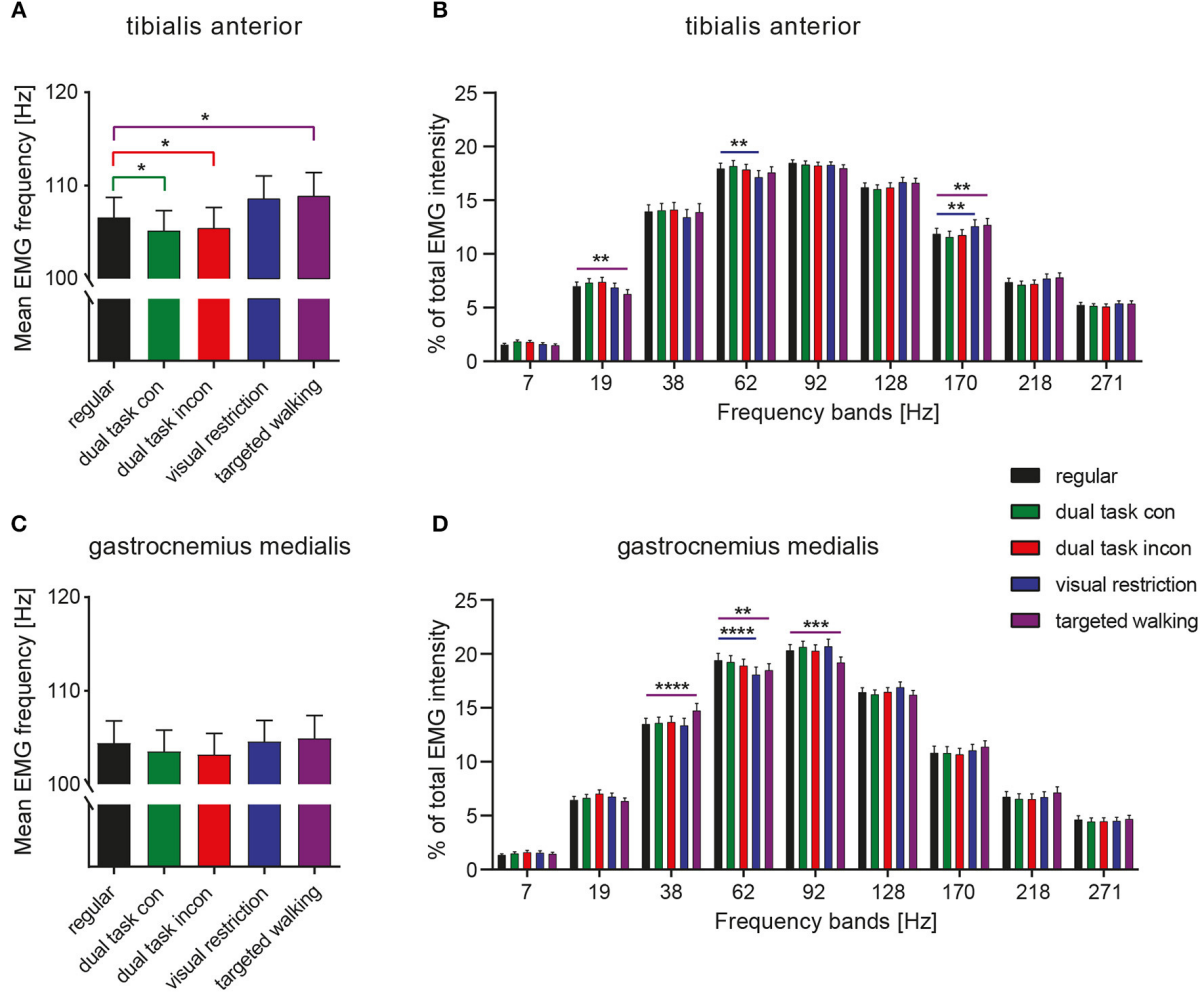
Spectral analysis of EMG intensity during different walking conditions. Mean EMG frequency of the TA **(A)** and GM muscle **(C)** during various locomotor tasks. Detailed spectral analysis of EMG intensity within different frequency bands of the TA **(B)** and GM muscle **(D)**. Statistical analysis of mean EMG frequencies during various tasks were performed by repeated measures 1-way ANOVA. Analysis of EMG intensity at specific frequency ranges was performed by repeated measures 2-way ANOVA with the independent factors frequency and locomotor conditions. Detailed effects of locomotor tasks on mean EMG frequency **(A,C)** and EMG intensity at different frequency bands **(B,D)** were examined by Dunnett's *post-hoc* correction for multiple comparisons. *P < 0.05; **P < 0.01; ***P < 0.001; ****P < 0.0001. con, congruent; incon, incongruent; EMG, electromyogram; GM, gastrocnemius medialis; TA, tibialis anterior.

The mean frequency of TA EMG intensity was changed by different locomotor tasks [Figure 2A; repeated measures 1-way ANOVA; *F*_(2.638, 129.3)_ = 9.963; *p* < 0.0001], whereas this was not observed in the GM [Figure 2C; *F*_(2.472, 93.95)_ = 1.344; *p* = 0.2663]. *Post-hoc* analysis revealed that mean frequency of TA EMG intensity was reduced during both dual task conditions (*p* = 0.0178 and *p* = 0.0411; repeated measures 1-way ANOVA followed by Dunnett's *post-hoc* test) but increased during targeted locomotion (*p* = 0.0482; Figure 2A). A repeated measures 2-way ANOVA was conducted on the influence of the two independent factors locomotor conditions and frequency bands on EMG intensity of TA and GM. There was no significant main effect of locomotor conditions [TA: *F*_(4, 196)_ = 1.0; *p* = 0.4087 and GM: *F*_(4, 152)_ = 0.9916; *p* = 0.4140], but of frequency [TA: *F*_(8, 392)_ = 170.4; *p* < 0.0001 and GM: *F*_(8, 304)_ = 173.6; *p* < 0.0001] on EMG intensity. The interaction effect was significant for both muscles [TA: *F*_(32, 1, 568)_ = 4.405; *p* < 0.0001 and GM: *F*_(32, 1, 216)_ = 3.785; *p* < 0.0001]. *Post-hoc* analysis revealed task-specific changes in EMG intensity within different frequency bands in the TA and GM. TA EMG intensity at low and intermediate frequencies was reduced, whereas TA intensity at higher frequencies was enhanced during both visual restriction and targeted walking conditions. Visual restriction resulted in reduced TA EMG intensity at 62 Hz (Figure 2B; *p* = 0.0021; repeated measures 2-way ANOVA followed by Dunnett's *post-hoc* test), whereas targeted walking induced reduced intensity at 19 Hz (*p* = 0.0074) compared to regular walking. Both visual restriction and targeted walking resulted in increased TA intensities at 170 Hz (visual restriction: *p* = 0.0082; targeted walking: *p* = 0.0015). Cognitive interference while walking did not alter the frequency characteristics of EMG intensity in the TA or GM (Figures 2B,D, **5**).

**Figure 3:**
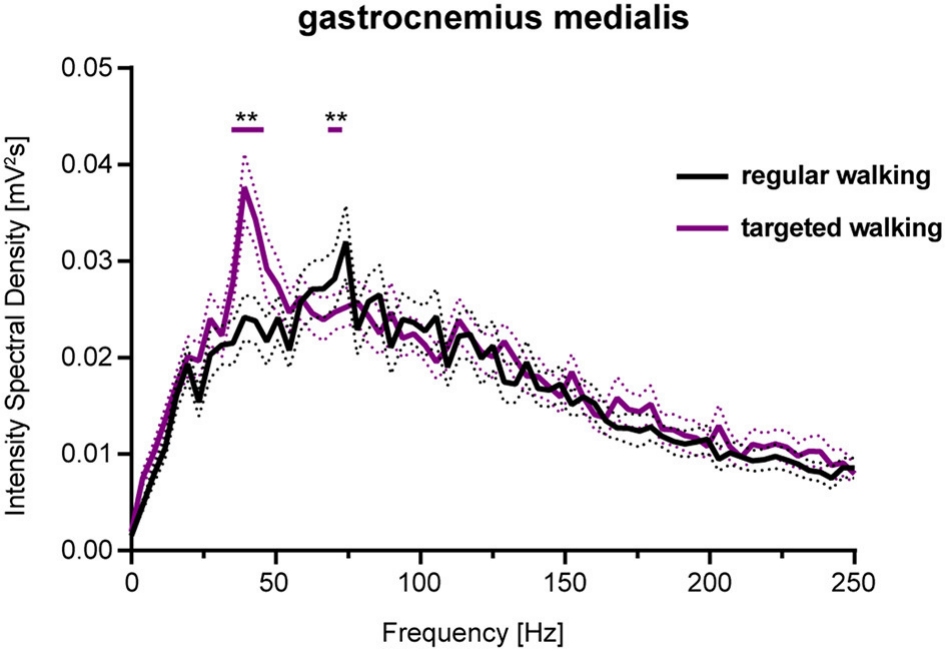
Spectral density of EMG intensity in GM during regular and targeted walking. EMG intensity of the GM muscle at different frequencies during regular (black line) and skilled, targeted walking (purple line). Data represent mean ± SEM of 27 healthy controls. Data were analyzed by repeated measures 2-way ANOVA with the independent factors frequency and locomotor conditions. Changes of EMG intensity at particular frequencies were examined by Dunnett's *post-hoc* test. **P < 0.01. EMG, electromyogram; GM, gastrocnemius medialis.

GM EMG intensity within specific frequency bands changed significantly during walking with partial visual restriction and targeted walking compared to regular locomotion (Figures 2D, **5**). EMG intensity at 62 Hz decreased with partial visual restriction (*p* = 0.0001; repeated measures 2-way ANOVA followed by Dunnett's *post-hoc* test; Figure 2D). Skilled, targeted walking led to enhanced EMG intensity at 38 Hz (*p* = 0.0001) and reduced intensity at 62 Hz (*p* = 0.0063) and 92 Hz (*p* = 0.0004). A Fourier based, high-resolution intensity spectral density plot revealing EMG intensity at various frequencies during targeted walking confirmed the enhanced GM intensity in the Piper rhythm range (35–47 Hz; *p* < 0.001; repeated measures 2-way ANOVA followed by Bonferroni's *post-hoc* test) and reduced intensity at 74 Hz (Figure 3; *p* < 0.001).

**Figure 4:**
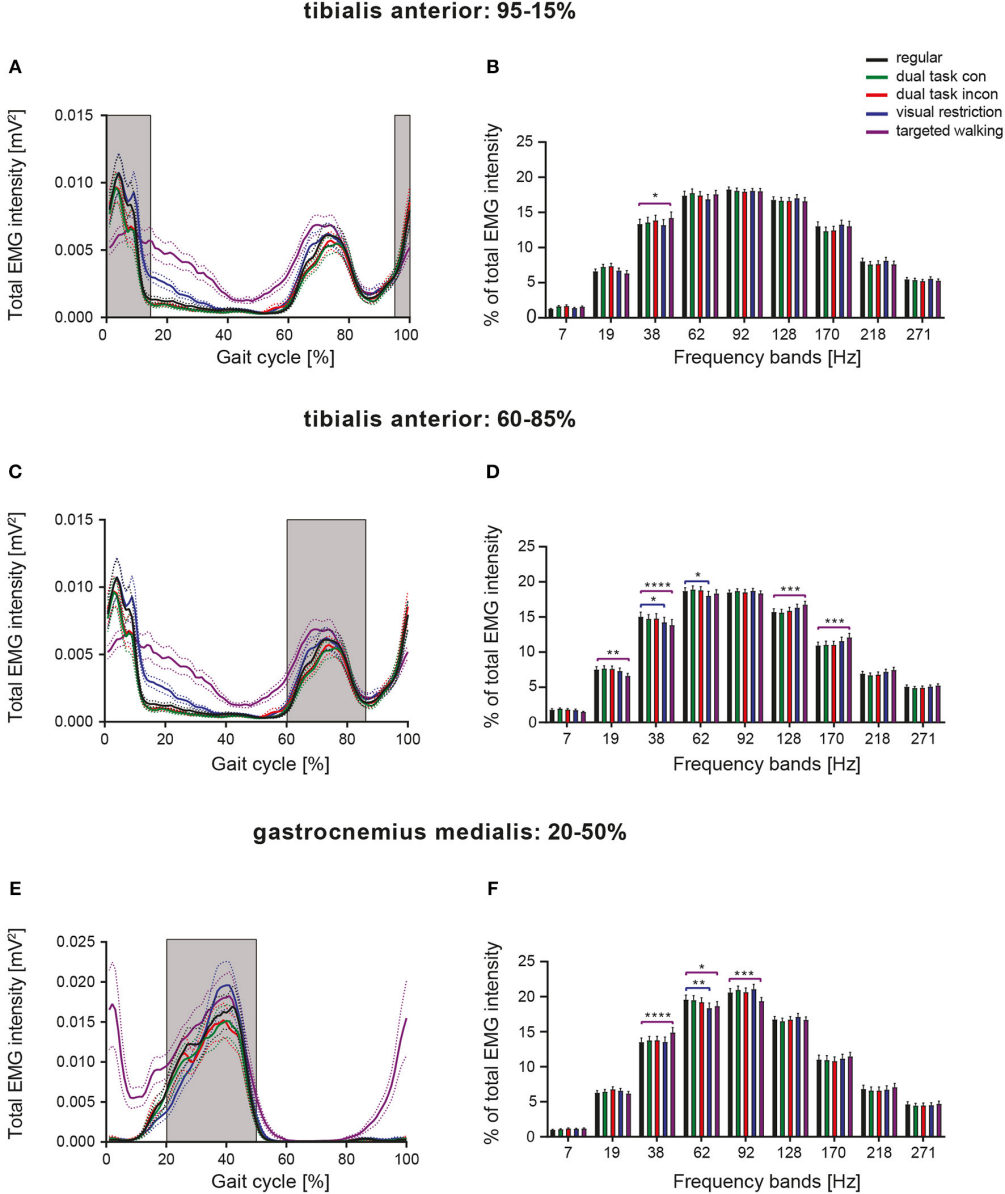
Phase-dependent modulation of neuromuscular control during different walking conditions. Averaged total EMG intensity and detailed EMG intensity at specific frequency bands over restricted activity periods of the TA [**(A,B)**: 95–15% of GC], [**(C,D)**: 60–85% of GC], as well as of the GM [**(E,F)**: 20–50% of GC]. Detailed analysis of EMG intensity at specific frequency ranges was performed by repeated measures 2-way ANOVA with the independent factors frequency and locomotor conditions. Task-specific changes in EMG intensity at particular frequency bands were examined by Dunnett's *post-hoc* test. *P < 0.05; **P < 0.01; ***P < 0.001; ****P < 0.0001. con, congruent; GC, gait cycle; incon, incongruent; EMG, electromyogram.

**Figure 5:**
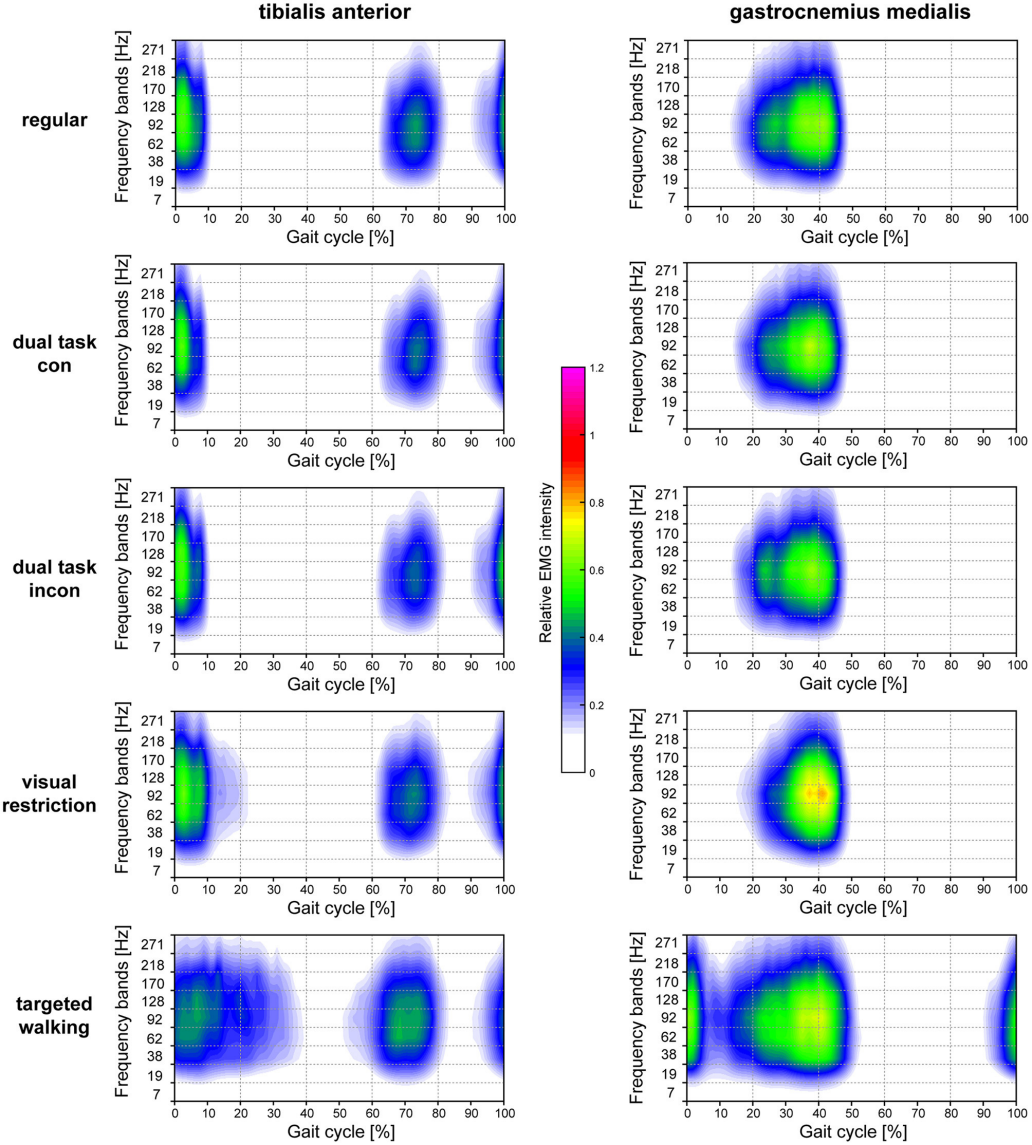
Heatmaps resolving time-amplitude-frequency characteristics of TA and GM EMGs during different walking conditions. Averaged EMG intensity over time and at specific frequencies during different locomotor tasks are displayed for the TA muscle (left column) and GM muscle (right column). EMG intensity is colored relative to the maximal EMG intensity measured during regular walking (maximal EMG intensity during regular walking corresponds to 1.0). con, congruent; incon, incongruent; EMG, electromyogram; GM, gastrocnemius medialis; TA, tibialis anterior.

To compare frequency characteristics of EMGs during different walking tasks in a more precise fashion, we assessed neuromuscular activity over limited periods, during which muscles were similarly active during all locomotor tasks (Figure 4). EMGs of TA were analyzed at 95–15 and 60–85% of the gait cycle (Figures 4A–D) corresponding to the mean activity phases of the muscle. A repeated measures 2-way ANOVA was conducted on the influence of the two independent factors locomotor conditions and frequency bands on EMG intensity. For both TA activity phases, there was a significant main effect of frequency bands [95–15%: *F*_(8, 392)_ = 141.4, *p* < 0.0001; 60–85%: *F*_(8, 392)_ = 175.2; *p* < 0.0001], but not of walking conditions [95–15%: *F*_(4, 196)_ = 2.117, *p* = 0.0801; 60–85%: *F*_(4, 196)_ = 0.7213; *p* = 0.5783; repeated measures 2-way ANOVA]. Significant interaction effects were found for both analyses [95–15%: *F*_(32, 1, 568)_ = 1.89, *p* = 0.0020; 60–85%: *F*_(32, 1, 568)_ = 3.844; *p* < 0.0001]. Depending on the respective phase within the gait cycle, we found different task-induced modifications of TA EMG intensity at particular frequencies, suggesting phase-dependent neural control of muscle function. TA activity during 95–15% of the gait cycle revealed enhanced EMG intensities at 38 Hz during visually guided, targeted walking (*p* = 0.0246; repeated measures 2-way ANOVA followed by Dunnett's *post-hoc* tests), whereas frequency modulations induced by partial visual restriction were not observed during this period of the gait cycle (Figure 4B). During 60–85% of the gait cycle, the EMG frequency pattern of the TA resembled that observed over the total gait cycle, with visual restriction resulting in reduced EMG intensity at 38 Hz (*p* = 0.0128) and at 62 Hz (*p* = 0.0465) and targeted walking leading to reduced EMG intensity at low frequencies (19 and 38 Hz; *p* = 0.0039, *p* = 0.0001) and enhanced intensity at high frequencies (128 and 170 Hz; *p* = 0.0008, *p* = 0.0001; Figure 4D). GM EMGs were assessed during 20–50% of the gait cycle (Figures 4E,F). There was a significant main effect of frequency bands [*F*_(8, 304)_ = 172.8, *p* < 0.0001; repeated measures 2-way ANOVA], but not of walking conditions [*F*_(4, 152)_ = 0.7699, *p* = 0.5464]. A significant interaction effect was observed [*F*_(32, 1, 216)_ = 3.344, *p* < 0.0001]. Task-specific changes in GM EMG frequency patterns closely resembled those observed over the total gait cycle (Figure 4F) and were mainly characterized by enhanced muscle intensity at 38 Hz as induced by skilled, targeted walking (*p* < 0.0001; repeated measures 2-way ANOVA followed by Dunnett's *post-hoc* tests: Figure 5).

### Effects of Walking Speed on Neuromuscular Control

**Figure 6:**
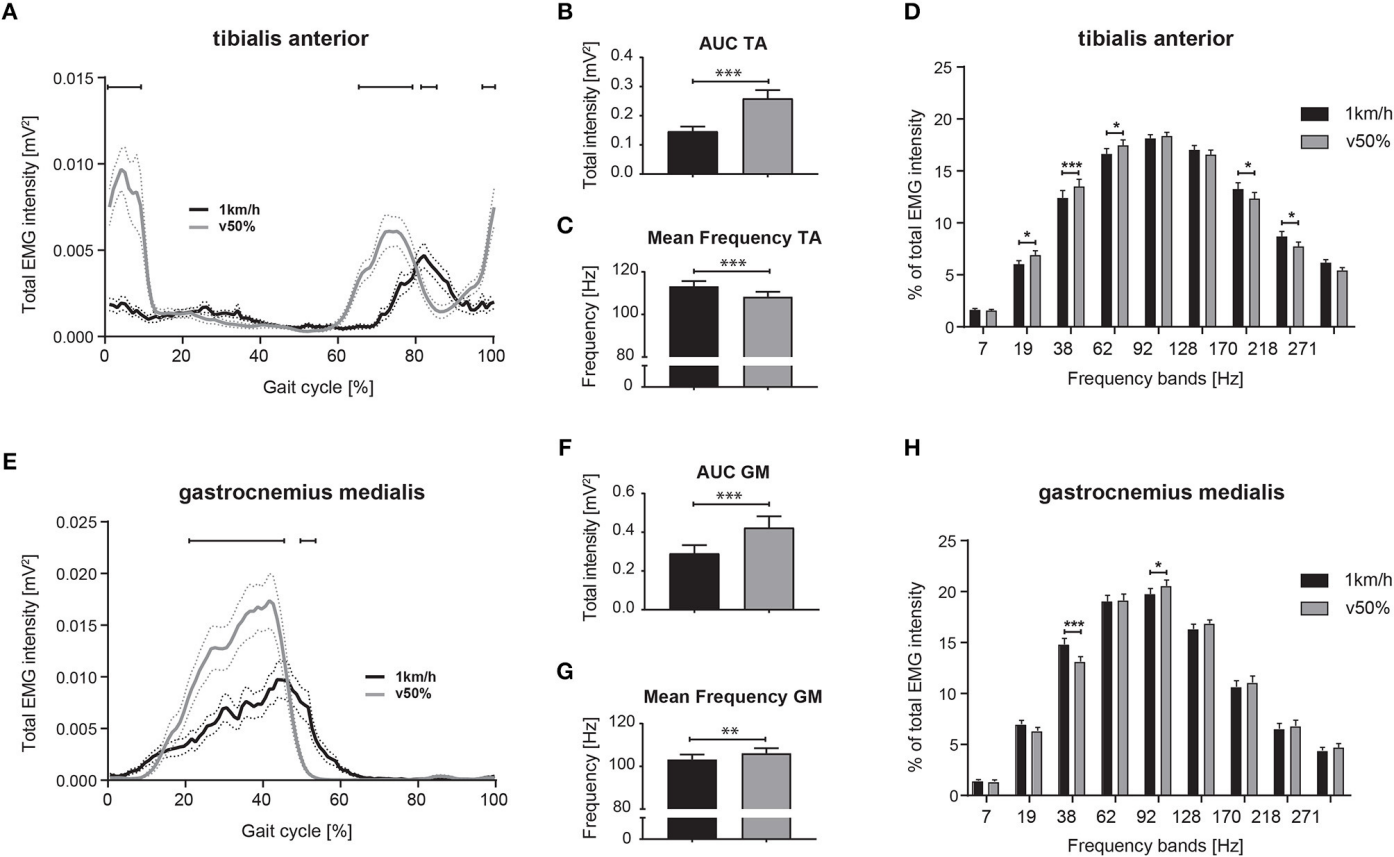
Differential neuromuscular control during slow and comfortable walking speed. Averaged total EMG intensity over time during slow (1 km/h) and half-maximal walking (v50%) speed for the TA **(A)** and GM muscle **(E)**. Area under the curve (AUC) representing EMG intensity and mean EMG frequency during slow and half-maximal gait velocity for the TA **(B,C)** and GM muscle **(F,G)**. EMG intensity at specific frequency bands during slow and faster walking for the TA **(D)** and GM muscle **(H)**. Statistical analysis of total EMG intensity over time and EMG intensity at specific frequency bands was performed using repeated measures 2-way ANOVA with the independent factors locomotor conditions and time (within gait cycle) or frequency bands, respectively. Changes of EMG intensity at particular time points **(A,E)** and frequencies **(D,H)** were examined by Bonferroni's *post-hoc* test. AUC and mean frequency data during slow and faster walking were analyzed with two-tailed, paired *t*-test. *P < 0.05; **P < 0.01; ***P < 0.001. AUC, area under the curve; EMG, electromyogram; GM, gastrocnemius medialis; TA, tibialis anterior; v50%, half-maximal walking speed.

EMGs were evaluated while regular walking at 1 km/h and at half-maximal walking speed (v_max50%_: 4.2 ± 0.5 km/h). EMGs of 4 subjects were excluded from this sub-analysis due to inadequate EMG recordings at the 1 km/h walking speed. Total EMG intensity of the distal leg muscles was substantially different during slow walking compared to v_max50%_ (Figures 6A,E). When analyzing total EMG intensity of TA and GM, we found significant main effects for the factor speed [TA: *F*_(1, 38)_ = 37.08; GM: *F*_(1, 36)_ = 20.44; *p* < 0.0001 for both; repeated measures 2-way ANOVA] and time within gait cycle [TA: *F*_(99, 3, 762)_ = 19.39; GM: *F*_(99, 3, 564)_ = 36.55; *p* < 0.0001 for both] with significant interaction effects [TA: *F*_(99, 3, 762)_ = 19.99; GM: *F*_(99, 3, 564)_ = 14.27; *p* < 0.0001 for both]. During slow walking, TA intensity was reduced at heel-strike (98–11% of gait cycle; *p* < 0.05; repeated measures 2-way ANOVA followed by Bonferroni's *post-hoc* test), while TA activation was delayed during initial swing phase (64–78 and 82–86% of gait cycle, *p* < 0.05). GM EMG intensity during slow walking was reduced during mid-stance (21–45% of gait cycle, *p* < 0.01) and revealed prolonged activity during late stance compared to v_max50%_ (Figure 6E; 49–54% of gait cycle; *p* < 0.05). Integrated EMG intensity (Figures 6B,F; AUC) was reduced in TA and GM during slow walking [paired, two-tailed *t*-test; TA: *t*_(38)_ = 5.89; GM: *t*_(36)_ = 4.54; *p* < 0.001 for both]. Slow walking speed resulted in enhanced mean frequency of TA EMG (Figure 6C; paired, two-tailed *t*-test; *t*_(38)_ = 5.06; *p* < 0.0001) and reduced mean frequency in GM EMG compared to v_max50%_ [Figure 6G; *t*_(36)_ = 3.15; *p* = 0.0034]. Interestingly, changes in the frequency domain of the EMG as induced by slow walking were similar to those observed during targeted walking (Figures 6D,H), i.e., slow walking led to reduced EMG intensity of the TA at low frequencies (19, 38, and 62 Hz; repeated measures 2-way ANOVA followed by Dunnett's *post-hoc* test) and to enhanced intensity at high frequencies (170, 218 Hz; Figure 6D). EMG intensity in the GM was enhanced at 38 Hz, and reduced at 92 Hz during slow walking (Figure 6H).

## Discussion

We demonstrated considerable task-dependent changes in neuromuscular control of ankle muscles as shown by time-amplitude and frequency resolved electromyography. Modifications of neuromuscular control were most pronounced while walking with visual restriction and during visually guided, targeted locomotion.

Walking during visual restriction resulted in more prolonged TA activity after initial foot contact and to delayed GM activation with enhanced peak EMG intensity. These changes in total EMG intensities compared to regular locomotion are likely associated with a shift toward reliance by the CNS on somatosensory and vestibular afferents during visual restriction resulting in adapted neuromuscular control (30–32).

Skilled, targeted walking diminished early TA intensity at foot contact and led to ectopic GM activity during late swing and early stance. These adaptations in distal leg EMGs suggest that, functionally, participants tried to hit the targets primarily with their forefoot, in keeping with our anecdotal observations. Enhanced total TA intensity during mid and late stance might be elicited by the challenging balance conditions during the single-limb phase, while the contralateral foot is being propelled toward the target (23). Premature and enhanced TA intensity during swing phase is in line with previous results on visually guided, targeted stepping (23, 38).

Contrary to visually restricted and targeted walking, dual task conditions induced only minor, and temporally limited changes in TA EMG intensity affecting primary and secondary muscle activity during early stance. Interestingly, cognitive dual tasks only modified the intensity but not the timing of TA activation.

There is convincing evidence that corticomuscular and intermuscular coherence in the range of 40 Hz indicates corticospinal drive to muscles during voluntary movements (13, 14, 17, 19, 20). Enhanced synchronization of motor unit action potentials in the Piper rhythm frequency was observed during highly challenging motor actions (22, 23), and were diminished in neurological conditions revealing impaired corticospinal integrity (25–27). In patients with incomplete spinal cord injury, neurorehabilitative training led to partial restoration of EMG intensity at Piper rhythm frequencies that was interpreted as training-induced recovery of corticospinal drive (21). Thus, spectral analysis of EMGs is a potential surrogate marker of pyramidal tract function (17, 19).

Enhanced EMG intensity at the Piper rhythm frequency that is indicative for additional cortical drive was observed in the GM during visually guided, targeted walking. Interestingly, increased EMG intensity of the TA at the Piper rhythm frequency around initial foot contact (95–15%) indicates a stronger neural drive during a gait phase that is known to be tightly controlled by supraspinal descending drive (10, 23, 39). Pronounced corticospinal control during visually-controlled challenging locomotion is consistent with preclinical (1, 3) and clinical studies (38). In contrast to an earlier study, we did not observe reduced EMG intensity at 38 Hz during dual task conditions (22). This discrepancy might be explained by the older population (mean age: 70.1 years) investigated by Clark et al. where cognitive interference likely induced stronger distraction of locomotor control and consequently resulted in more pronounced gait deviations (30). Moreover, walking speed between the different tasks was not kept constant in the study by Clark and colleagues (leading to velocity-dependent changes in gait pattern) and the different cognitive dual tasks used in the two studies (auditory 2-back test vs. Stroop tests) might pose different types or degrees of challenge to participants. We did not find differential effects of the additional cognitive load required by the incongruent vs. the congruent Stroop tests on TA and GM EMGs. It is possible that the dual task conditions may not have been sufficiently challenging to induce major deviations in the EMGs of distal leg muscles in our population.

Slow walking at 1 km/h induced major changes in the timing of muscle activity brought about by the reduced foot roll dynamics and delayed initiation of swing compared to walking at a more comfortable pace (40, 41). Interestingly, changes in the frequency characteristics of EMGs during slow walking resembled those elicited by targeted walking. These results support earlier literature suggesting that slow speed is more dependent on precisely timed supraspinal inputs, most likely due to the higher stability requirements of slow walking (26, 42, 43). Walking at 1 km/h on a treadmill is unusual and uncomfortable for most healthy individuals, whereas walking at half-maximal speed more closely resembles the preferred movement pattern and is performed under more autonomous control.

The present study suggests that corticospinal drive to the distal leg musculature becomes more relevant during demanding walking tasks as control shifts along a continuum from spinal to more supraspinal influence. Similarly, recent studies have shown that complex walking tasks requiring challenging ankle joint control also result in enhanced EMG intensity in the Piper frequency band (22, 38, 44). It was demonstrated that intermuscular EMG synchrony of distal agonist muscles (soleus and GM muscle) reveals sufficient sensitivity to detect changes in corticospinal drive during different locomotor tasks (22, 38). Specifically, Piper rhythm synchrony increased during a more challenging walking task that share some similarities with the targeted walking performed in our study, thus supporting our interpretation of enhanced corticospinal drive during demanding locomotor conditions.

Neural drive not only concerns cortico-muscular control but may also include the selection of active muscle fiber types. According to Wakeling and Rozitis, changes in EMG intensity in high frequency regions of the spectra, as observed here in the TA during visual restriction and targeted walking, suggest a higher proportion of fast conducting muscle fibers being activated during these tasks (28). The CNS appears able to select between different muscle fiber types to control particular movements (20, 45). The control and fine-tuning of complex, targeted movements requires not only force but also the augmentation of more subtle muscle properties. This neuromuscular adaptation can be visualized using highly resolved time-frequency analysis of EMG intensity.

Time-frequency analysis of TA EMGs revealed that task-specific modulations of neuromuscular control differed depending on the gait phase. Phase-dependent modulation of supraspinal and spinal drive during different time points in the gait cycle have been described in earlier studies (39, 46). Enhanced EMG intensity in TA at 38 Hz during heel strike is in agreement with reports consisting of increased supraspinal control during this period of the gait cycle (10, 39).

A limitation of this study is that we analyzed EMGs of antagonistic muscles only and thus were not able to perform EMG coherence analysis. The lack of coherence analysis with simultaneous electroencephalography (EEG) or EMG in other muscles does not allow the direct linking of EMG intensity at particular frequencies to descending neural drive. Hence, we rely on reports from the literature supporting the concept that EMG frequencies in the region of 30–60 Hz primarily originate from descending cortical inputs (21, 22, 26, 38, 47). Moreover, corticospinal drive was assessed by electromyography rather than by direct measurements of cortical neuronal activity. Thus, changes in EMG intensity at different frequencies may not only have been induced by neural inputs, but also by peripheral factors such as thickness of subcutaneous tissue or muscle fiber properties (10, 11, 48). However, different walking tasks were performed during a short, 30 min protocol and using the same setup (fixed walking speed, electrodes positioned at the same place etc.), thus minimizing these peripheral effects as far as possible. Another limitation is that we only analyzed EMGs of distal leg muscles, although voluntary foot placement during walking is also dependent on the activity of proximal muscles such as the hip abductors that were not considered in this study (49, 50).

## Conclusion

Here we demonstrate task-specific and phase-dependent modulations of neuromuscular control as measured by changes in the time, amplitude, and frequency characteristics of EMGs. The most pronounced changes in neuromuscular control were induced by visually guided, targeted walking, during which we observed enhanced EMG intensity in TA and GM in the Piper frequency band. These results suggest enhanced cortical contributions to movement control during challenging sensorimotor conditions. Decoding the role of different CNS networks in controlling various forms of motor programs is important for developing customized rehabilitation programs that aim to target particular CNS systems. Moreover, monitoring EMG intensity in the Piper frequency band might enable to assess recovery of corticospinal drive in response to physical training or other interventions in neurological patients.

## Data Availability Statement

All analyzed datasets for this study are included in the manuscript and the supplementary files.

## Author Contributions

LF, ML, AC, MB, and BZ contributed to the conception or design of the work. LF, CM, LL, VvT, BG, and BZ contributed to the acquisition, analysis, or interpretation of data for the work. LF performed the statistical analysis. LF, TK, VvT, and BZ contributed to drafting the work or revising it critically for important intellectual content. All authors approved the final version of the manuscript and agree to be accountable for all aspects of the work. All authors listed on the manuscript meet the criteria for authorship as requested by the journal.

### Conflict of Interest Statement

The authors declare that the research was conducted in the absence of any commercial or financial relationships that could be construed as a potential conflict of interest.
